# Supplementary figures and images for: Global burden of subarachnoid hemorrhage among adolescents and young adults aged 15–39 years: A trend analysis study from 1990 to 2021
Source: PLoS One. 2024 Dec 20;19(12):e0316111. doi: 10.1371/journal.pone.0316111 (PMC11661650; doi:10.1371/journal.pone.0316111)

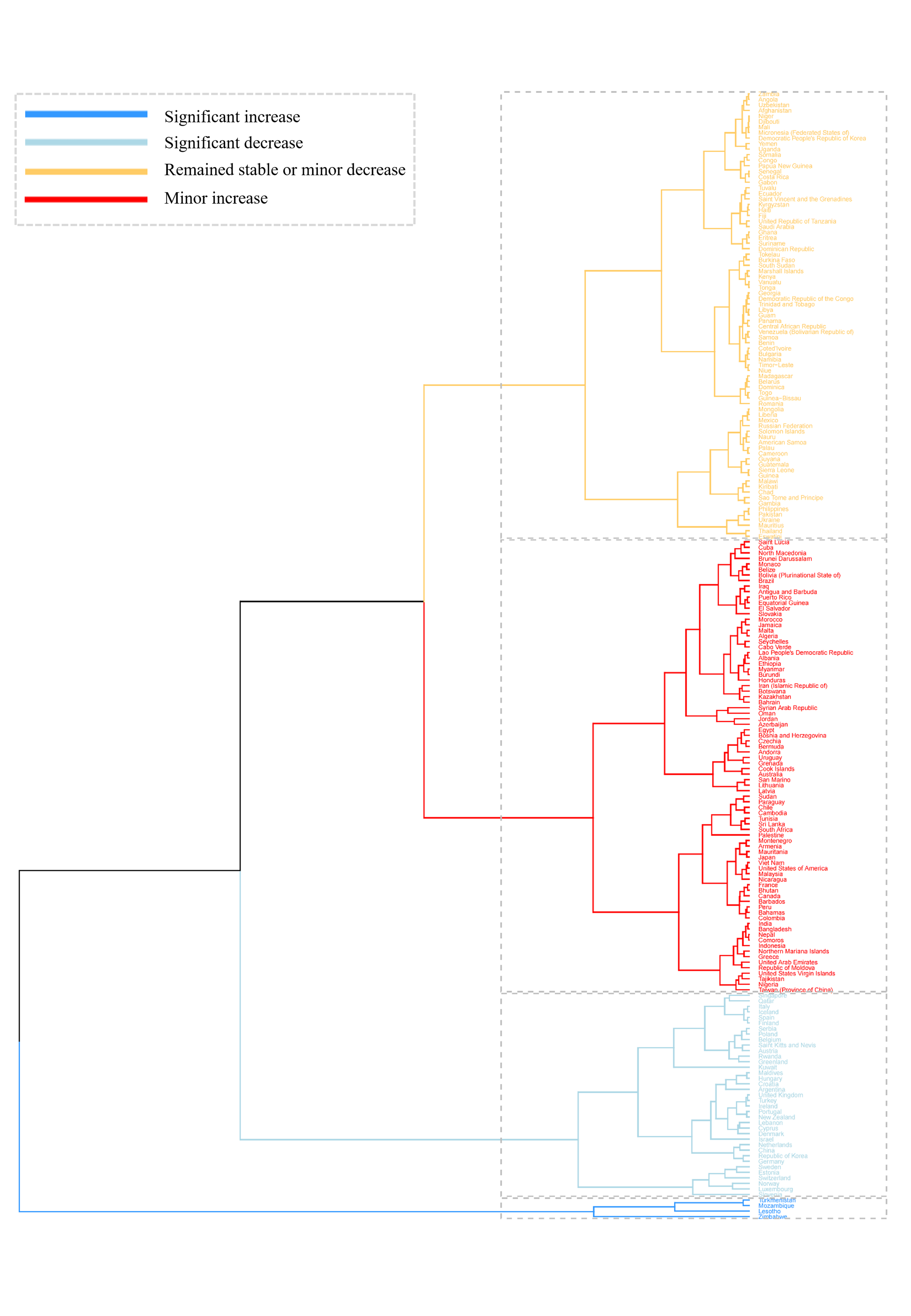

Supplement: S1 Fig — (TIF) [file pone.0316111.s007.tif]
