# Supplementary material for: Global burden of subarachnoid hemorrhage among adolescents and young adults aged 15–39 years: A trend analysis study from 1990 to 2021
Source: PLoS One. 2024 Dec 20;19(12):e0316111. doi: 10.1371/journal.pone.0316111 (PMC11661650; doi:10.1371/journal.pone.0316111)
Supplement: Attachment — Submitted filename: Response to Reviewers.docx [file pone.0316111.s008.docx]

**4 September 2024**

Muhammad Fawad, PhD

Academic Editor

PLOS ONE

Dear Editor:

We really appreciate the critical reading of our manuscript entitled “*Global burden of subarachnoid hemorrhage among adolescents and young adults aged 15–39 years: A trend analysis study from 1990 to 2021*” (Manuscript Number: PONE-D-24-29086R1). We thank you for all the valuable suggestions received from the reviewers and editor. We have carefully considered the comments and have revised the manuscript accordingly. The responses to the comments are listed one by one as follows (please see below). We are looking forward to hearing about your decision.

**Journal Requirements:**

1. When submitting your revision, we need you to address these additional requirements.

Please ensure that your manuscript meets PLOS ONE's style requirements, including those for file naming. The PLOS ONE style templates can be found at 
https://journals.plos.org/plosone/s/file?id=wjVg/PLOSOne_formatting_sample_main_body.pdf and 
<https://journals.plos.org/plosone/s/file?id=ba62/PLOSOne_formatting_sample_title_authors_affiliations.pdf>

**Answer:** Thank you for your comments. I have made the necessary formatting revisions as per your requirements and have resubmitted the manuscript.

2. Thank you for stating in your Funding Statement: 
This work was financially supported by the Basic Research Plan of Shanxi Province in 2021, grant number 202103021224386, awarded to Dr. Hongming Ji. The funding body played no role in the study design, data collection, data analysis, decision to publish, or preparation of the manuscript. For more information about the funding source, please visit the official website at https://kjt.shanxi.gov.cn/.

Please provide an amended statement that declares all the funding or sources of support (whether external or internal to your organization) received during this study, as detailed online in our guide for authors at http://journals.plos.org/plosone/s/submit-now.  Please also include the statement “There was no additional external funding received for this study.” in your updated Funding Statement. 
Please include your amended Funding Statement within your cover letter. We will change the online submission form on your behalf.

**Answer:** Thank you for your correction regarding the accuracy of my Funding Statement. I have now uploaded a revised version that includes additional details about other sources of financial support received during this study.

*This work was funded by the Basic Research Plan of Shanxi Province in 2021 (202103021224386) and the 2024 Graduate Education Innovation Plan of Shanxi Province (2024SJ177). The funders had no role in study design, data collection and analysis, decision to publish, or preparation of the manuscript. There was no additional external funding received for this study.*

3. When completing the data availability statement of the submission form, you indicated that you will make your data available on acceptance. We strongly recommend all authors decide on a data sharing plan before acceptance, as the process can be lengthy and hold up publication timelines. Please note that, though access restrictions are acceptable now, your entire data will need to be made freely accessible if your manuscript is accepted for publication. This policy applies to all data except where public deposition would breach compliance with the protocol approved by your research ethics board. If you are unable to adhere to our open data policy, please kindly revise your statement to explain your reasoning and we will seek the editor's input on an exemption. Please be assured that, once you have provided your new statement, the assessment of your exemption will not hold up the peer review process.

Answer: All relevant data are within the manuscript and its Supporting Information files.

4. PLOS requires an ORCID iD for the corresponding author in Editorial Manager on papers submitted after December 6th, 2016. Please ensure that you have an ORCID iD and that it is validated in Editorial Manager. To do this, go to ‘Update my Information’ (in the upper left-hand corner of the main menu), and click on the Fetch/Validate link next to the ORCID field. This will take you to the ORCID site and allow you to create a new iD or authenticate a pre-existing iD in Editorial Manager. Please see the following video for instructions on linking an ORCID iD to your Editorial Manager account: <https://www.youtube.com/watch?v=_xcclfuvtxQ>

**Answer:** Thank you for your guidance. I have now uploaded the ORCID iD for the corresponding author and completed the necessary information update.

5. Ethics statement appears in the Methods section of the manuscript AND at the end of the manuscript:
   Your ethics statement should only appear in the Methods section of your manuscript. If your ethics statement is written in any section besides the Methods, please delete it from any other section.

**Answer:** Thank you for your feedback regarding the placement of the ethics statement. I have now moved the ethics statement to the Methods section of the manuscript.

6. We note that Figure S1 in your submission contain map images which may be copyrighted. All PLOS content is published under the Creative Commons Attribution License (CC BY 4.0), which means that the manuscript, images, and Supporting Information files will be freely available online, and any third party is permitted to access, download, copy, distribute, and use these materials in any way, even commercially, with proper attribution. For these reasons, we cannot publish previously copyrighted maps or satellite images created using proprietary data, such as Google software (Google Maps, Street View, and Earth). For more information, see our copyright guidelines: http://journals.plos.org/plosone/s/licenses-and-copyright.
    
   We require you to either present written permission from the copyright holder to publish these figures specifically under the CC BY 4.0 license, or remove the figures from your submission:
    
   a. You may seek permission from the original copyright holder of Figure S1 to publish the content specifically under the CC BY 4.0 license.  
    
   We recommend that you contact the original copyright holder with the Content Permission Form (http://journals.plos.org/plosone/s/file?id=7c09/content-permission-form.pdf) and the following text:
   “I request permission for the open-access journal PLOS ONE to publish XXX under the Creative Commons Attribution License (CCAL) CC BY 4.0 (http://creativecommons.org/licenses/by/4.0/). Please be aware that this license allows unrestricted use and distribution, even commercially, by third parties. Please reply and provide explicit written permission to publish XXX under a CC BY license and complete the attached form.”
    
   Please upload the completed Content Permission Form or other proof of granted permissions as an "Other" file with your submission.
    
   In the figure caption of the copyrighted figure, please include the following text: “Reprinted from [ref] under a CC BY license, with permission from [name of publisher], original copyright [original copyright year].”
    
   b. If you are unable to obtain permission from the original copyright holder to publish these figures under the CC BY 4.0 license or if the copyright holder’s requirements are incompatible with the CC BY 4.0 license, please either i) remove the figure or ii) supply a replacement figure that complies with the CC BY 4.0 license. Please check copyright information on all replacement figures and update the figure caption with source information. If applicable, please specify in the figure caption text when a figure is similar but not identical to the original image and is therefore for illustrative purposes only.
   The following resources for replacing copyrighted map figures may be helpful:
    
   USGS National Map Viewer (public domain): http://viewer.nationalmap.gov/viewer/
   The Gateway to Astronaut Photography of Earth (public domain): http://eol.jsc.nasa.gov/sseop/clickmap/
   Maps at the CIA (public domain): https://www.cia.gov/library/publications/the-world-factbook/index.html and https://www.cia.gov/library/publications/cia-maps-publications/index.html
   NASA Earth Observatory (public domain): http://earthobservatory.nasa.gov/
   Landsat: http://landsat.visibleearth.nasa.gov/
   USGS EROS (Earth Resources Observatory and Science (EROS) Center) (public domain): http://eros.usgs.gov/#
   Natural Earth (public domain): <http://www.naturalearthdata.com/>

**Answer:** Thank you for your comments and for emphasizing the importance of map copyright. Considering the copyright concerns and the variations in national boundaries across different countries and regions, as well as the fact that the original Figure S1 was supplementary to Table 1 in the manuscript, we have decided to remove Figure S1. This removal does not alter the main discussion of the manuscript, and the remaining tables can adequately provide the necessary detailed data.

7. Please review your reference list to ensure that it is complete and correct. If you have cited papers that have been retracted, please include the rationale for doing so in the manuscript text, or remove these references and replace them with relevant current references. Any changes to the reference list should be mentioned in the rebuttal letter that accompanies your revised manuscript. If you need to cite a retracted article, indicate the article’s retracted status in the References list and also include a citation and full reference for the retraction notice.

**Answer:** We have carefully reviewed and verified that our reference list does not include any retracted papers. The references we removed have been marked in red, and the new references we added have been highlighted in blue.

**Additional Editor Comments:**

Authors should provide more insights into the specific socio-economic or healthcare factors that might help clarify why Middle-SDI regions experience a higher burden than Low-SDI regions?

**Answer:** Thank you for your suggestion regarding the discussion. I have added a discussion on the specific characteristics and higher burden in Middle-SDI regions compared to Low-SDI and High-SDI countries, with a focus on their unique socio-economic factors and healthcare challenges.

Manuscripts Page 21-22 Line 354--369

*“Our analysis reveals that Middle-SDI regions exhibited higher incidence and prevalence rates of SAH compared to Low-SDI regions, corroborating the findings of Lv B et al. regarding the greater SAH disease burden in Middle-SDI regions**[15].* *This observation can be attributed to a complex interplay of factors characteristic of these transitioning economies.* *Middle-SDI regions are undergoing a rapid epidemiological transition, marked by evolving healthcare systems and significant lifestyle changes. While these areas have developed better diagnostic capabilities than their low-SDI counterparts, they often lack the advanced treatment facilities and specialized care available in high-SDI regions [16].* *This disparity in healthcare infrastructure may lead to increased detection of SAH cases without commensurate improvement in treatment outcomes, potentially contributing to higher prevalence rates.* *Concurrently, these regions are experiencing rapid urbanization, which has precipitated substantial lifestyle changes.* *The urban environment often fosters increased psychosocial stress, more sedentary behaviors, and the adoption of unhealthy dietary habits - all recognized risk factors for SAH* *[17].* *”*

Did the authors consider the impact of gender-specific healthcare access and health-seeking behaviors when explaining the observed disparities between men and women?

**Answer:** Thank you for your valuable feedback. The impact of gender-specific healthcare access and health-seeking behaviors was indeed an aspect that was previously lacking in our discussion. I have now expanded the discussion in the revised manuscript to include a more detailed analysis of the differences in healthcare access and health-seeking behaviors between men and women.

Manuscripts Page 22 Line 369--383

*“The confluence of these urbanization-related factors may exacerbate the incidence of SAH in these transitioning economies. Gender disparities further complicate this landscape. While women generally demonstrate a higher propensity for seeking preventive healthcare services [18], potentially facilitating earlier detection and management of SAH risk factors, they may also face significant sociocultural barriers to accessing healthcare in certain contexts. In some societies, women may prioritize family care responsibilities over personal health needs [19]. These contrasting dynamics create a complex interplay that influences gender-specific SAH patterns in middle-SDI regions. The combination of transitioning healthcare systems, urbanization-related lifestyle changes, and gender-specific factors thus presents a unique challenge in addressing the SAH burden in these evolving economies. This complex scenario likely contributes to the observed higher incidence and prevalence rates in middle and low-middle SDI regions, highlighting the need for targeted interventions that address these multifaceted challenges.*

The figures in the manuscript are blurry and difficult to interpret. Please improve the resolution of these figures or consider redrawing them to ensure a clear presentation and easy understanding for readers.

**Answer:** Thank you for your feedback. I have redrawn all the figures in the manuscript to meet the journal's requirements and ensure they are clearly visible for readers. However, please note that the PDF version generated by the Editorial Manager system may render the images less clear. To view or download high-resolution versions of the figures, you can click on the "Click here to access/download" link in the top right corner of each image. We apologize for any inconvenience this may cause.

**Comments to the Author**

1. Is the manuscript technically sound, and do the data support the conclusions?

The manuscript must describe a technically sound piece of scientific research with data that supports the conclusions. Experiments must have been conducted rigorously, with appropriate controls, replication, and sample sizes. The conclusions must be drawn appropriately based on the data presented.

Reviewer #1: Yes

Reviewer #2: Yes

Reviewer #3: Yes

2. Has the statistical analysis been performed appropriately and rigorously?

Reviewer #1: Yes

Reviewer #2: Yes

Reviewer #3: Yes

3. Have the authors made all data underlying the findings in their manuscript fully available?

The [PLOS Data policy](http://www.plosone.org/static/policies.action#sharing) requires authors to make all data underlying the findings described in their manuscript fully available without restriction, with rare exception (please refer to the Data Availability Statement in the manuscript PDF file). The data should be provided as part of the manuscript or its supporting information, or deposited to a public repository. For example, in addition to summary statistics, the data points behind means, medians and variance measures should be available. If there are restrictions on publicly sharing data—e.g. participant privacy or use of data from a third party—those must be specified.

Reviewer #1: Yes

Reviewer #2: Yes

Reviewer #3: Yes

4. Is the manuscript presented in an intelligible fashion and written in standard English?

PLOS ONE does not copyedit accepted manuscripts, so the language in submitted articles must be clear, correct, and unambiguous. Any typographical or grammatical errors should be corrected at revision, so please note any specific errors here.

Reviewer #1: Yes

Reviewer #2: Yes

Reviewer #3: Yes

**Answer:** We sincerely appreciate the reviewers' and editor's recognition of our data handling and the content of the manuscript. On behalf of all the authors, I would like to thank you for your thorough review and constructive feedback. We will carefully revise the manuscript according to your professional and insightful comments. Once again, thank you for your expert review and valuable input.

5. Review Comments to the Author

**Reviewer #1****:** Strengths: The study has a clear and specific objective: analyzing the global burden of subarachnoid hemorrhage (SAH) among adolescents and young adults (AYAs) aged 15–39 years over a 31-year period, focusing on spatial and temporal trends. This provides a targeted approach, which is beneficial for creating specialized public health strategies.
Opportunities for Improvement: The objective could be enhanced by mentioning the geographical scope and the importance of the study in the context of global health explicitly. Additionally, the rationale for selecting the age group 15-39 years should be briefly explained to highlight its significance.

**Answer:** Thank you for highlighting the strengths of our study and for your valuable suggestions. In response to your feedback, we have explicitly emphasized the rationale for selecting the AYAs population in the manuscript. We have noted that this age group has unique lifestyle habits and risk factors, making them a critical demographic for targeted public health strategies. Additionally, we have expanded the discussion to underscore the global health relevance of our study, emphasizing its geographical scope and the importance of understanding SAH trends in this context.

***Line 90-99***

*“****This age group faces unique challenges, including the onset of independent living, educational pursuits, and career establishment, which can influence health behaviors and access to care[8]. Understanding the burden of SAH in this age group is critical for designing effective prevention and treatment programs that address the specific needs of AYAs.***

*Our study utilizes the 2021 Global Burden of Disease (GBD) database, providing the most comprehensive and current estimates of subarachnoid hemorrhage (SAH) burden across all 204 countries and territories[9]. Unlike previous studies limited to pre-2019 data, this research offers post-COVID-19 insights and focuses specifically on the adolescent and young adults population. ”*

Methods
Strengths:
The study utilizes data from the Global Burden of Disease Study 2021, which is a comprehensive and widely recognized source.
Segmenting the data by age groups and socio-demographic index (SDI) quintiles allows for a detailed analysis of different populations.
The use of statistical methods such as Joinpoint regression and decomposition analysis adds robustness to the analysis of temporal trends and epidemiological changes.
Opportunities for Improvement:
 The methods section could benefit from a more detailed explanation of the statistical techniques used, particularly Joinpoint regression and decomposition analysis, to aid readers who may not be familiar with these methods.
Including a brief discussion on data limitations, such as potential biases or gaps in the GBD 2021 data, would provide a more balanced view of the study's methodology.
**Answer:** Thank you for your thoughtful feedback on the Methods section. In response to your suggestions, we have added a more detailed explanation of the statistical techniques used, particularly Joinpoint regression and decomposition analysis. These additions are intended to assist readers who may not be familiar with these methods. We have also included relevant references to further support the explanation of these techniques.

*Line 148-157*

*“**Joinpoint regression is a statistical method used to analyze trends in time-series data, such as the incidence, prevalence, mortality, and DALYs. This method partitions the continuous time-series data's linear regression model into several statistically significant trend segments, with each portion described by a linear model to identify the optimal number of turning points. The method uses a Monte Carlo permutation test to determine the optimal number of joinpoints and their locations based on the statistical significance of the change in trend[12]. For each segment, the Joinpoint regression model calculates the annual percentage change (APC) in the outcome variable and the 95% confidence intervals for the APC. The average annual percentage change (AAPC) is calculated over the entire study period.”*

*Line 160-170*

*“**To quantify the drivers of changes in the number of individuals with SAH, we employed decomposition analysis to estimate the relative contributions of population growth, aging, and epidemiological change. This method, developed by Das Gupta and widely applicable in epidemiological research, offers a principled dissection of how each component uniquely contributes to the overall trend[13]. It summarizes the contribution of various factors to observed changes by algebraically isolating the standardized impact of each contributing multiplicative factor. Epidemiological change involves changes in the incidence or prevalence of disease due to various factors, such as improvements in healthcare, the introduction or evolution of risk factors, the effectiveness of prevention strategies, advancements in treatment, or changes in environmental or lifestyle factors.”*

Results
Strengths:
The results are presented with clear numerical data, showing both absolute numbers and age-standardized rates, which enhances the comprehensiveness of the analysis.
Highlighting the decrease in mortality and DALYs alongside the increase in incidence and prevalence provides a nuanced understanding of the burden of SAH among AYAs.
The regional analysis adds valuable insights into where the burden is most substantial, which is crucial for targeted interventions.
Opportunities for Improvement:
The results section could be improved by including visual aids such as graphs or charts to illustrate trends more effectively.
Providing a more in-depth analysis of why certain trends occurred, especially the increase in incidence and prevalence in recent years, would add depth to the findings.

**Answer:** Thank you for your thoughtful feedback on the Results section. We appreciate your recognition of the strengths in our presentation of the numerical data and regional analysis. In response to your suggestions, we have expanded the Discussion section to provide a more in-depth analysis of the observed trends, particularly the increase in incidence and prevalence in recent years. We have also enhanced Table 1 by including the percentage change in values from 1990 to 2021, which provides a more intuitive understanding of the data trends.

*Line 264 Table 1*

*Line 354-383*

*Our analysis reveals that Middle-SDI regions exhibited higher incidence and prevalence rates of SAH compared to Low-SDI regions, corroborating the findings of Lv B et al. regarding the greater SAH disease burden in Middle-SDI regions[15]. This observation can be attributed to a complex interplay of factors characteristic of these transitioning economies. Middle-SDI regions are undergoing a rapid epidemiological transition, marked by evolving healthcare systems and significant lifestyle changes. While these areas have developed better diagnostic capabilities than their low-SDI counterparts, they often lack the advanced treatment facilities and specialized care available in high-SDI regions [16]. This disparity in healthcare infrastructure may lead to increased detection of SAH cases without commensurate improvement in treatment outcomes, potentially contributing to higher prevalence rates. Concurrently, these regions are experiencing rapid urbanization, which has precipitated substantial lifestyle changes. The urban environment often fosters increased psychosocial stress, more sedentary behaviors, and the adoption of unhealthy dietary habits - all recognized risk factors for SAH [17]. The confluence of these urbanization-related factors may exacerbate the incidence of SAH in these transitioning economies. Gender disparities further complicate this landscape. While women generally demonstrate a higher propensity for seeking preventive healthcare services [18], potentially facilitating earlier detection and management of SAH risk factors, they may also face significant sociocultural barriers to accessing healthcare in certain contexts. In some societies, women may prioritize family care responsibilities over personal health needs [19]. These contrasting dynamics create a complex interplay that influences gender-specific SAH patterns in middle-SDI regions. The combination of transitioning healthcare systems, urbanization-related lifestyle changes, and gender-specific factors thus presents a unique challenge in addressing the SAH burden in these evolving economies. This complex scenario likely contributes to the observed higher incidence and prevalence rates in middle and low-middle SDI regions, highlighting the need for targeted interventions that address these multifaceted challenges.*

Discussion and Conclusion
Strengths:
The discussion appropriately emphasizes the need for targeted public health interventions in low and low-middle SDI regions.
The conclusion ties the findings to practical recommendations, such as improving healthcare resources and preventive strategies, making the study's implications clear and actionable.
Opportunities for Improvement:
The discussion could benefit from a comparison with other studies or existing literature to place the findings within a broader context.
Expanding on potential future research directions or unanswered questions that arose from the study would provide a pathway for further investigation and enhance the overall impact of the research.

**Answer:** Thank you for your valuable feedback on the Discussion and Conclusion sections. Following your suggestions, we have expanded the discussion to include a comparison with other studies and existing literature, placing our findings within a broader context. Specifically, we have added a discussion on Middle SDI regions and considered the implications of our findings in the post-COVID-19 era, which broadens our understanding of the specific populations studied.

*Line 490-496*

*“Our study is uniquely positioned within the critical timeframe encompassing the COVID-19 pandemic, extending the analysis beyond 2019. This temporal context is crucial, as it allows us to examine SAH trends against the backdrop of unprecedented global health challenges. Furthermore, our research specifically targets adolescent populations, an age group often underrepresented in SAH studies. By focusing on this demographic during such a pivotal period, we address a significant gap in the existing literature.*

*In conclusion, our study's distinct combination of adolescent focus and post-2019 data provides essential insights for developing targeted SAH prevention strategies and epidemiological policies. These findings are particularly valuable in the context of long COVID, offering crucial guidance for mitigating SAH risks in young populations as we navigate the evolving post-pandemic landscape.”*

Overall Assessment
The study provides a comprehensive analysis of the global burden of subarachnoid hemorrhage among adolescents and young adults, with clear strengths in data utilization, statistical analysis, and practical recommendations. However, there are opportunities to improve the clarity and depth of the methodology, results, and discussion sections. Enhancing these aspects would strengthen the study's contribution to public health knowledge and policy formulation.

**Answer:** Thank you for your overall assessment and for recognizing the strengths of our study in terms of data utilization, statistical analysis, and practical recommendations. We appreciate your constructive feedback on improving the clarity and depth of the methodology, results, and discussion sections. In response to your suggestions, we have carefully revised these sections to enhance their precision and comprehensiveness. We believe these improvements will further strengthen the study’s contribution to public health knowledge and policy formulation. We are grateful for your valuable input and the opportunity to refine our work.

**Reviewer #2:** I appreciate the opportunity to critically review the manuscript PONE-D-24-29086, in which the authors analyze the burden of SAH among adolescents and young adults using the most recent GBD data (2021). The manuscript is clear, and the methodology is robust. I present a series of minor comments for the authors' consideration.

Comment 1: Lines 370-371: The authors indicate that they found the burden of SAH to be higher in men. While this is true, the current wording makes it appear that this is a unique finding of the study, which is not correct. There is extensive published evidence indicating that the burden of cardiovascular diseases is generally lower among women. I suggest briefly mentioning this.

**Answer:** Thank you for your correction. You are absolutely right, and it was an oversight in our writing process. We have deleted the statement on Lines 370-371, as indicated in the revised manuscript at Line 434. Additionally, we have expanded the subsequent discussion to include an analysis of the specific reasons for the lower incidence among women, as supported by previous research.

*Line 441-450*

*“Fuentes AM's study demonstrates a higher prevalence of aneurysms and SAH among older females, suggesting that postmenopausal estrogen decline may increase aneurysm risk. Our research on premenopausal women epidemiologically validates estrogen's protective effect against aneurysm formation, offering insights into hormonal influences on SAH risk across women's life stages[41]. Estrogen's protective role in the cardiovascular system is attributed to its promotion of nitric oxide (NO) production, which maintains vascular elasticity and function while reducing inflammatory responses and oxidative stress[42]. These findings underscore the significance of sex hormones in SAH risk assessment and prevention strategies for women at different ages. ”*

Comment 2: Published evidence (DOI: 10.1016/j.jacc.2023.11.007) suggests that metabolic risk factors (i.e., high systolic blood pressure) and dietary factors have a significant attributable fraction in SAH, and in general (as expected) this was higher in older age groups. Although the authors did not aim to analyze risk factors, it would be interesting if they briefly discussed the impact these risk factors might have on this particular age group, composed of young individuals.

**Answer:** Thank you for your valuable suggestion. Your recommendation to consider the impact of risk factors has indeed enriched the depth and significance of our discussion. Following your advice, we have included a discussion on the complex influence of metabolic risk factors on younger populations and have also addressed how dietary factors may affect blood pressure and cardiovascular risk in this age group.

*Line 415-428*

*“Wh**en examining specific youth populations,* *George A. Mensah's research reveals that metabolic risk factors, particularly high systolic blood pressure, and dietary factors significantly contribute to SAH etio**logy, with a more pronounced impact on the elderly**[33].* *However, for younger demographics, lifestyle patterns emerge as more critical determinants.* *Beneficial dietary habits, regular physical activity, and adequate sleep are linked to lower systolic blood pressure in younger individuals**[34].* *Yet, factors such as smoking, unhealthy food choices, and stress increase their susceptibility to cardiovascular diseases**[35]. This paradox adds complexity to understanding SAH risk in youth.* *In terms of diet, the intak**e of fruits and vegetables among adolescents is generally insufficient, while the consumption of carbonated soft drinks and fast food increases the risk of cardiovascular diseases[36]. These factors underscore the importance of focusing on the unique lifestyle patterns of younger populations and highlight the urgency of developing appropriate public health strategies to address these issues.*

*Our study found that the burden of SAH is higher in men, which is consistent with extensive published evidence indicating that the burden of cardiovascular diseases, including stroke, is generally lower among women**[37].* *This gender disparity has been well-documented in the literature, and our findings align with these established trends.”*

Comment 3: Figures 1-3 were illegible. I would appreciate it if they could be presented clearly should I have the pleasure of being considered as a reviewer for the revised version of the document.

**Answer:** Thank you for your feedback regarding the figures, and we apologize for the previous issues with legibility. We have improved the clarity and resolution of Figures 1-3 to ensure they are presented clearly. However, please note that the PDF version generated by the Editorial Manager system may still render the images less clear. To view or download high-resolution versions, you can click on the "Click here to access/download" link in the top right corner of each image. We apologize for any inconvenience this may cause and hope these adjustments make the figures easier to interpret.

Comment 4: The GBD 2021 Forecasting Collaborators Group conducted analyses up to the year 2050. Is there any particular methodological reason for conducting the analysis up to the year 2040? I suggest specifying this in the Discussion section.

**Answer:** Thank you for your insightful comment regarding the forecasting timeframe. We chose to conduct our analysis up to the year 2040, rather than 2050, due to several considerations. The GBD 2021 database includes data from the COVID-19 pandemic period (2019-2021), during which we observed significant shifts in key metrics such as incidence rates. We were concerned that the uncertainties and potential inaccuracies in data collection during the pandemic might introduce bias into longer-term forecasts. Therefore, to minimize potential forecasting bias due to these data anomalies, we opted for a shorter forecasting period ending in 2040. This approach is consistent with other published studies, such as the one with DOI: 10.1016/S2468-2667(23)00122-6, which also chose 2040 as the cutoff year for similar reasons. We have now included a detailed explanation of this decision in the Discussion section of our manuscript. We look forward to future updates in the GBD database, which we hope will provide more accurate post-pandemic data, enabling us to extend our forecasts with greater confidence. Once again, thank you for your attention to detail and for helping to enhance the credibility of our work.

*Line 475-489*

*“Our predictions are based on GBD data collected before and during the COVID-19 pandemic (2019-2021)[47], introducing potential biases due to the pandemic's impact on non-communicable diseases and healthcare systems[48]. The pandemic's economic consequences may have increased psychological stress among young people, potentially raising SAH incidence rates[49]. Additionally, isolation policies often prevented timely access to critical medical interventions[50]. Given these considerations, we limited our forecast to 2040, acknowledging the uncertainty introduced by recent pandemic-related data. As our understanding of COVID-19 and its aftermath evolves, future GBD updates may enable more accurate long-term predictions. Our projections suggest that without intervention, SAH incidence among AYAs will increase over the next 20 years. This underscores the urgent need for further research, focusing on both early clinical screening and tertiary prevention. As SAH continues to pose a significant neurosurgical burden, a multifaceted approach to prevention, early detection, and treatment is essential to mitigate its impact on AYAs in the coming decades.”*

**Reviewer #3:** This is a manuscript with interesting title that written well.
There are only some minor mistakes in some lines (including 197, 262, …) that can be corrected.
Also the quality of figures are very low that need to improve.

**Answer:** Thank you for your thorough review and constructive feedback on our manuscript. We acknowledge the minor oversights on our part and have revised line 197 for more accurate wording. We also removed the map in line 262 due to potential disputes arising from differing national boundary definitions. Since the map was primarily for data visualization, its removal does not affect the overall content or the regional discussion in the manuscript. Additionally, we have improved the quality of the figures as requested. However, please note that the PDF version generated by the Editorial Manager system may still render the images less clear. To view or download high-resolution versions, you can click on the "Click here to access/download" link in the top right corner of each image. We apologize for any inconvenience this may cause and appreciate your careful review and valuable suggestions.

*Line 207*

*“The age-standardized incidence rate (ASIR) exhibited an AAPC of -0.80% (95% CI: -0.85%, -0.75%), while the age-standardized prevalence rate (ASPR) demonstrated an AAPC of -0.65% (95% CI: -0.66%, -0.64%) over the study period.”*

I suggest that in the decomposition analysis, the clustering analysis was performed and the characteristics of clusters across on the SDI categories, region, … was assessed.

**Answer:** Thank you for your professional and insightful suggestion. We have taken your advice and reanalyzed the decomposition data, incorporating a detailed clustering analysis across the SDI categories and regions. The results of this analysis have been added to the S3 table, and we have expanded the Results section to include a more comprehensive discussion of the contributions of various factors across different SDI regions. We appreciate your valuable feedback, which has significantly enhanced the depth of our analysis.

*Line 288-289*

*“ The growth in global prevalence exhibited a pattern similar to that of incidence* ***(Fig 2B)****. ”*

*Line 292-293*

*“Similarly, the reduction in global DALYs mirrored the pattern observed in deaths* ***(Fig 2D)****.”*

*Line 294-300*

*“Among the five SDI regions, High and High-middle SDI regions demonstrate an overall decrease in incidence, prevalence, deaths, and DALYs, primarily attributable to epidemiological improvements. Conversely, Low-middle and Low SDI areas exhibit increases in all metrics, mainly due to population growth. Middle SDI regions present a nuanced picture: slight increases in incidence and prevalence driven by population growth, yet decreases in deaths and DALYs owing to epidemiological advancements****(S3 table)****. ”*
