# Supplementary material for: Global burden of subarachnoid hemorrhage among adolescents and young adults aged 15–39 years: A trend analysis study from 1990 to 2021
Source: PLoS One. 2024 Dec 20;19(12):e0316111. doi: 10.1371/journal.pone.0316111 (PMC11661650; doi:10.1371/journal.pone.0316111)
Supplement: Attachment — Submitted filename: Response to Reviewers.docx [file pone.0316111.s009.docx]

**31 October 2024**

Muhammad Fawad, PhD

Academic Editor

PLOS ONE

Dear Editor:

We really appreciate the critical reading of our manuscript entitled “*Global burden of subarachnoid hemorrhage among adolescents and young adults aged 15–39 years: A trend analysis study from 1990 to 2021*” (Manuscript Number: PONE-D-24-29086R1). We thank you for all the valuable suggestions received from the reviewers and editor. We have carefully considered the comments and have revised the manuscript accordingly. The responses to the comments are listed one by one as follows (please see below). We are looking forward to hearing about your decision.

Journal Requirements:

Please review your reference list to ensure that it is complete and correct. If you have cited papers that have been retracted, please include the rationale for doing so in the manuscript text, or remove these references and replace them with relevant current references. Any changes to the reference list should be mentioned in the rebuttal letter that accompanies your revised manuscript. If you need to cite a retracted article, indicate the article’s retracted status in the References list and also include a citation and full reference for the retraction notice.

**Answer:** We have carefully reviewed and verified that our reference list does not include any retracted papers. The references we removed have been marked in red, and the new references we added have been highlighted in blue.

Additional Editor Comments:

The authors' response effectively addresses both the reviewer's and my comments; however, some concerns from the reviewer still remain, and these should be clarified.

**Answer:** Thank you for your constructive feedback and for the reviewers' insightful comments. I have carefully revised the manuscript based on the initial feedback and have made further modifications to address the remaining concerns.

Reviewers' comments:

Reviewer's Responses to Questions

Comments to the Author

1. If the authors have adequately addressed your comments raised in a previous round of review and you feel that this manuscript is now acceptable for publication, you may indicate that here to bypass the “Comments to the Author” section, enter your conflict of interest statement in the “Confidential to Editor” section, and submit your "Accept" recommendation.

Reviewer #1: All comments have been addressed

Reviewer #2: All comments have been addressed

Reviewer #3: (No Response)

2. Is the manuscript technically sound, and do the data support the conclusions?

The manuscript must describe a technically sound piece of scientific research with data that supports the conclusions. Experiments must have been conducted rigorously, with appropriate controls, replication, and sample sizes. The conclusions must be drawn appropriately based on the data presented.

Reviewer #1: Yes

Reviewer #2: Yes

Reviewer #3: Yes

3. Has the statistical analysis been performed appropriately and rigorously?

Reviewer #1: Yes

Reviewer #2: Yes

Reviewer #3: Yes

4. Have the authors made all data underlying the findings in their manuscript fully available?

The PLOS Data policy requires authors to make all data underlying the findings described in their manuscript fully available without restriction, with rare exception (please refer to the Data Availability Statement in the manuscript PDF file). The data should be provided as part of the manuscript or its supporting information, or deposited to a public repository. For example, in addition to summary statistics, the data points behind means, medians and variance measures should be available. If there are restrictions on publicly sharing data—e.g. participant privacy or use of data from a third party—those must be specified.

Reviewer #1: Yes

Reviewer #2: Yes

Reviewer #3: Yes

5. Is the manuscript presented in an intelligible fashion and written in standard English?

PLOS ONE does not copyedit accepted manuscripts, so the language in submitted articles must be clear, correct, and unambiguous. Any typographical or grammatical errors should be corrected at revision, so please note any specific errors here.

Reviewer #1: Yes

Reviewer #2: Yes

Reviewer #3: Yes

**Answer:** We sincerely appreciate the reviewers' and editor's recognition of our data handling and manuscript content. Thank you for your thorough review and constructive feedback. We will revise the manuscript in line with your insightful comments. Once again, thank you for your valuable input.

6. Review Comments to the Author

Reviewer #1: The article presents interesting results of great importance for public health. The authors have made the necessary changes. The article can be accepted for publication.

**Answer:** We are grateful to Reviewer #1 for recognizing the significance of our results for public health and for acknowledging the improvements made to the manuscript. Thank you for recommending our work for publication.

Reviewer #2: I thank the group of authors for considering the observations and suggestions I previously provided. I congratulate them on their interesting manuscript. It was a pleasure to critically review it.

**Answer:** We sincerely thank Reviewer #2 for their thoughtful observations and kind words. It was our pleasure to incorporate your valuable suggestions.

Reviewer #3: Unfortunately, there is no correction for the clustering analysis in the manuscript.

The aim of the clustering analysis is to detect homogenous subgroups of countries without considering the SDI categories.

**Answer:** Thank you very much for your constructive suggestion. We apologize for not fully understanding the application of the clustering method initially. After your emphasis, we extensively reviewed relevant literature to gain a better understanding of this method. Consequently, we have incorporated a Hierarchical Cluster Analysis to assess the AAPC of deaths and DALYs across the 21 regions and 204 countries and territories mentioned in our study. This analysis aims to identify different patterns of disease burden changes. We have included the clustering results for the 21 regions in Figure 2 of the main text, and due to space constraints, the clustering of the 204 countries and territories is provided in Supplementary Figure S1. Additionally, we attempted to explain the different clustering patterns and have included detailed AAPC data in the supplementary materials. The Statistical Analysis section now describes our methodology. Since the primary focus of our paper is the overall global trends of adolescent SAH, we have utilized Hierarchical Cluster Analysis to broadly categorize similar patterns of change. We appreciate your suggestion and recognize that using cluster analysis in GBD database studies can open new research avenues. We will pay more attention to this clustering method to detect homogenous subgroups of countries without considering SDI categories in future research. Thank you once again for your invaluable input.

*Line 160-167*

*“The AAPC values for deaths and DALYs were estimated using a linear regression model. Hierarchical cluster analysis was then performed based on the AAPC values to evaluate the patterns of disease burden changes across 21 regions and 204 countries and territories. At the regional level, all regions were classified into four categories: remained stable or minor decrease, moderate decrease, substantial decrease, and significant decrease. At the country level, the 204 countries and territories were similarly categorized into four groups: significant increase, minor increase, remained stable or minor decrease, and significant decrease.”*

*Line 283-301*

## *****“Cluster*** *Analysis***

***To identify regions with similar patterns of change in SAH burden among the AYA population, a hierarchical cluster analysis was performed across 21 regions. The regions were classified into four categories based on the AAPC in rates for SAH-related deaths and DALYs. Eastern Europe and Southern Sub-Saharan Africa demonstrated a trend of remained stable or minor decrease. In contrast, regions such as Western Europe, Southern Latin America, and East Asia exhibited a significant decrease, indicating a marked reduction in the SAH burden* ***(Fig 2, S3 table)****. Notably, among the 204 countries and territories, Zimbabwe, Lesotho, Mozambique, and Turkmenistan exhibit a significant increase in SAH burden* ***(S1 Fig, S4 table)****.***

***Fig 2. Cluster analysis results based on the AAPC values of the age-standardized rates for deaths and DALYs attributed to SAH burden in the global AYA population from 1990 to 2021.***

***Notes:*** *This figure presents the hierarchical clustering of regions based on the AAPC in age-standardized rates for deaths and DALYs attributed to SAH. Regions are classified into four categories: remained stable or minor decrease (blue), moderate decrease (light blue), substantial decrease (yellow), and significant decrease (red).*

***Abbreviations:*** *AAPC: estimated annual percentage change; DALYs: disability-adjusted-life-years.”*
